# Anomalous Decay of Nanomechanical Modes Going Through Nonlinear Resonance

**DOI:** 10.1038/s41598-017-17184-6

**Published:** 2017-12-22

**Authors:** O. Shoshani, S. W. Shaw, M. I. Dykman

**Affiliations:** 1 Ben-Gurion University of the Negev, Beer-Sheva, 8410501 Israel; 2 Florida Institute of Technology, Melbourne, FL 32901 USA; 3 Michigan State University, East Lansing, MI 48824 USA

## Abstract

Because of the small size of nanomechanical systems, their vibrations become nonlinear already for small amplitudes. Many nontrivial aspects of the vibration dynamics arise from the coexistence of several nonlinearly coupled modes. We show that such coupling can lead to anomalous decay of the modes where they go through nonlinear resonance, so that their amplitude-dependent frequencies become commensurate. We demonstrate the possibility of a strongly nonmonotonic dependence of the decay rate on the amplitude if one of the modes serves as a thermal reservoir for another mode. Where the decay of both modes is slow compared to the rate of resonant energy exchange, the decay is accompanied by amplitude oscillations. Depending on the initial conditions, with increasing time it can display an extremely sharp or a comparatively smooth crossover between different regimes. The results provide insight into recent experimental results by several groups and suggest new ways of characterizing and controlling nanomechanical systems.

## Introduction

The nonlinear resonance occurs where two vibrational frequencies in the system are commensurate, i.e., their ratio is a rational number. The resonance effects are most pronounced where both the numerator and the denominator of the corresponding fraction are comparatively small integers, for example, where one of the frequencies is twice or three times the other. The study of nonlinear resonance has a long history in quantum and classical mechanics. It goes back at least to Laplace and Poincare on the classical side and to the Fermi resonance on the quantum side^1,2^. The resonance has been observed in a broad range of systems, from celestial bodies to ecological systems to molecules^3–6^. Recently, nonlinear resonance has attracted particular interest in the context of nano- and micro-mechanical vibrational systems^7–15^ and microwave cavities used in quantum information^16,17^. These mesoscopic systems provide unprecedented access to studying, using, and controlling this complicated phenomenon.

In conservative classical systems, nonlinear resonance leads to energy oscillations between the resonating modes. This is reminiscent of the energy oscillations between two coupled harmonic oscillators with close frequencies. However, the actual picture in nonlinear resonance is more complicated, extending to dynamical chaos. On the quantum side, nonlinear resonance is in some sense simpler in the absence of dissipation, as its primary signature is the familiar level repulsion.

The quantum situation changes if the resonating modes are dissipative. If the modes have very different decay rates, one of them can serve as a thermal reservoir for another, cf.^18^. This effect has been used to drive a slowly decaying microwave cavity mode to a coherent quantum state^16^; it extends to driven modes^19^, and such extension has attracted much attention in cavity optomechanics^20^.

An important advantageous feature of mesoscopic oscillators is the possibility to tune them in and out of nonlinear resonance. This can be done by directly controlling their frequencies^7^ or dynamically, using the dependence of the frequency on the vibration amplitude. Here, by driving a mode, one brings its overtone into resonance with an overtone of another mode, which is then also excited. The ensuing backaction significantly changes the dynamics of the driven mode and, in particular, its decay after the driving is switched off. Such decay is a major means of studying mesoscopic vibrational systems^21^.

In this paper we develop a theory of the decay of classical vibrational modes brought into nonlinear resonance. We reveal the rich and unusual pattern of the decay. In particular, if the decay rates of the involved modes are significantly different, the fast decaying mode can be an efficient thermal reservoir for a slowly decaying mode where the modes resonate. Since the mode frequencies depend on their amplitudes, the resonance is transient. This leads to a characteristic peak in the instantaneous decay rate of the slowly decaying mode as a function of time. The effect can be thought of as a transient classical analog of the well-known in quantum physics Purcell effect^22^.

The resonant dynamics is very different if the decay rates of both modes are smaller than their nonlinear resonant coupling in the appropriate units. In this case decay is accompanied by comparatively fast energy exchange between the modes that leads to oscillations of the vibration amplitudes. Because the strength of the nonlinear mode coupling strongly depends on the amplitudes, the oscillations are qualitatively different from those in linear resonance. We develop a general framework for analyzing the decay in this situation. It reveals the qualitative features of the decay, including sharp or smooth crossovers between different regimes with varying time. It also allows one to establish the range of parameters and the initial conditions where different types of behavior occur. The approach relies on the existence of a broad parameter range where, as we show, nonlinear dynamics in the absence of decay is much simpler than that in the general Poincare picture of nonlinear resonance in conservative systems^1,23^.

## Results

### A minimalistic model

**Figure 1:**
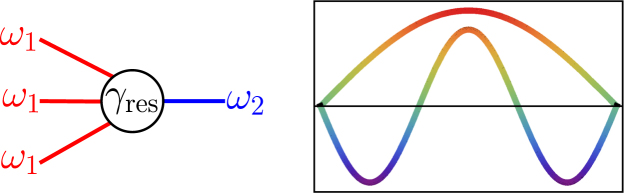
Schematic of 3:1 resonance. Left panel: three excitations (quanta) of mode 1 with frequency *ω*_1_ can resonantly scatter into an excitation (quantum) of mode 2 with frequency *ω*_2_ ≈ 3*ω*_1_, and vice versa, an excitation of mode 2 can resonantly scatter into three excitations of mode 1; *γ*_res_ is the parameter of the resonant mode coupling. Right panel: a sketch of the first and third flexural modes in a stretched nanowire; these modes are close to 3:1 resonance.

To be specific, we will consider the modes with the frequency ratio close to 3:1, as sketched in Fig. 1. For symmetry reasons, the coupling between such modes in nano- and micromechanical systems is often stronger than the coupling between the modes close to 2:1 resonance. The interesting recent work^14,15^, which has been done in parallel with the present paper, reports the observations of 3:1 resonance and the rich dynamics that come with it.

We describe the modes by the Duffing model, that is conventionally used in nanomechanics^24–26^, complemented by the term that accounts for the resonant nonlinear mode coupling. The Hamiltonian of the system reads

1 $$H=\sum _{n}\,[\frac{1}{2}({p}_{n}^{2}+{\omega }_{n}^{2}{q}_{n}^{2})+\frac{1}{4}{\gamma }_{n}{q}_{n}^{4}]+{\gamma }_{{\rm{res}}}{q}_{1}^{3}{q}_{2}\mathrm{.}$$

Here, *q*_*n*_ and *p*_*n*_ are the coordinate and momentum of mode *n* (*n* = 1, 2), *ω*_1,2_ and *γ*_1,2_ are the mode eigenfrequencies and the Duffing nonlinearity parameters, and *γ*_res_ is the mode coupling parameter. The model (1) captures the essential features of the resonant behavior.

Nonlinear resonance in nanomechanics is most easily observed where the mode nonlinearity is weak in the sense that the quartic in *q*_*n*_ terms in *H* are small compared to the quadratic terms. Therefore 3:1 resonance happens for 3*ω*_1_ close to *ω*_2_, so that the frequency difference *δω*_12_ = 3*ω*_1_ − *ω*_2_ is small, $|\delta {\omega }_{12}|\,\ll \,{\omega }_{\mathrm{1,2}}$. Then the modes can be tuned into exact resonance by varying their vibration amplitudes *A*_1,2_ and using that the effective vibration frequency of an *n*th mode is ${\omega }_{n}^{{\rm{eff}}}\approx {\omega }_{n}+3{\gamma }_{n}{A}_{n}^{2}\mathrm{/8}{\omega }_{n}$ ^27^. Typically, in the experiment it is the low-frequency mode that is directly excited and tuned into the nonlinear resonance, cf.^7,8,14,15^.

For small $|\delta {\omega }_{12}|$, the amplitude range of interest is where the amplitude-dependent frequency change is small compared to *ω*_1,2_. This significantly simplifies the analysis, as one can use the rotating wave approximation (RWA) and change from the rapidly oscillating coordinates and momenta of the modes to new scaled coordinates and momenta, ${q}_{n}={\omega }_{n}^{-\mathrm{1/2}}({Q}_{n}\,\cos \,{{\varphi }}_{n}+{P}_{n}\,\sin \,{{\varphi }}_{n}),\,{p}_{n}=-{\omega }_{n}^{\mathrm{1/2}}({Q}_{n}\,\sin \,{{\varphi }}_{n}-{P}_{n}\,\cos \,{{\varphi }}_{n})$ with *ϕ*_1_ = *ω*_1_ *t* and *ϕ*_2_ = 3*ω*_1_ *t*. Functions *Q*_*n*_, *P*_*n*_ remain almost constant over time 1/*ω*_1_,1/*ω*_2_. The equations of motion for *Q*_*n*_, *P*_*n*_ are

2 $${\dot{Q}}_{n}=-{{\rm{\Gamma }}}_{n}{Q}_{n}+\frac{\partial {H}_{{\rm{RWA}}}}{\partial {P}_{n}},\quad {\dot{P}}_{n}=-{{\rm{\Gamma }}}_{n}{P}_{n}-\frac{\partial {H}_{{\rm{RWA}}}}{\partial {Q}_{n}},$$

where *H*_RWA_ is the RWA Hamiltonian,

3 $${H}_{{\rm{RWA}}}=-\frac{1}{2}\delta {\omega }_{12}({Q}_{2}^{2}+{P}_{2}^{2})+\sum _{n}\frac{3{\gamma }_{n}}{32{\omega }_{n}^{2}}{({Q}_{n}^{2}+{P}_{n}^{2})}^{2}+\frac{{\gamma }_{{\rm{res}}}}{8\sqrt{3}{\omega }_{1}^{2}}{\rm{R}}e\,[({Q}_{1}-i{P}_{1}{)}^{3}({Q}_{2}+i{P}_{2}\mathrm{)].}$$

Whereas the terms with *H*_RWA_ in Eq. (2) come directly from the Hamiltonian (1), the terms $\propto {{\rm{\Gamma }}}_{\mathrm{1,2}}$ have been added to account for mode decay. They come from the linear friction forces ${-}2{{\rm{\Gamma }}}_{n}{\dot{q}}_{n}$ experienced by nanomechanical modes. With *δω*_12_ = 3*ω*_1_ *−* *ω*_2_, the Hamiltonian *H*_RWA_ does not contain terms quadratic in *Q*_1_, *P*_1_. The nonlinear terms disregarded in (3) either renormalize the parameters or lead to corrections that do not change the dynamics qualitatively. Here we aim at revealing most interesting characteristic features of this resonant dynamics.

### Resonant transient nonlinear friction

Along with the linear friction force, nanomechanical modes often experience nonlinear friction, where the friction coefficient depends on the mode amplitude^28–34^. The microscopic mechanisms considered so far predict that the friction coefficient either monotonically increases^18,35,36^, or decreases^33,37^, with the increasing mode amplitude. In contrast, resonant mode coupling can lead to an anomalously strong nonlinear friction and nonmonotonic amplitude dependence of the friction coefficient.

In systems of coupled nano- and micro-mechanical modes, the higher-frequency modes often decay faster than the lower-frequency ones^8,10,38,39^. In the case of nonlinear resonance, if the higher-frequency mode 2 decays much faster than mode 1, Γ_2_ $\gg$ Γ_1_, mode 2 can serve as a thermal reservoir for mode 1. This reservoir is special, as it has a finite bandwidth ∼Γ_2_. Therefore it is most efficient only when the amplitude-dependent frequency detuning $3{\omega }_{1}^{{\rm{eff}}}-{\omega }_{2}^{{\rm{eff}}}$ is within this bandwidth. In addition, the coupling to the reservoir is nonlinear in the mode-1 coordinate, which also makes the decay rate of this mode amplitude-dependent.

Other conditions needed for mode 2 to serve as a thermal reservoir for mode 1 and the derivation of the equation of motion for mode 1 are given in Methods. It is convenient to write this equation for a dimensionless complex amplitude of mode 1 𝒜 ${\mathcal{A}}={\mathrm{(3}{\gamma }_{1}\mathrm{/8}{\omega }_{1}^{2}{{\rm{\Gamma }}}_{2})}^{\mathrm{1/2}}({Q}_{1}-i{P}_{1})\,\exp [-i{\rm{\Phi }}]$, where Φ(*t*) is the phase that accumulates due to the amplitude dependence of the mode frequency, $\dot{{\rm{\Phi }}}(t)={{\rm{\Gamma }}}_{2}|{\mathcal{A}}(t{)|}^{2}$,

4 $$\mathop{<mml:mpadded xmlns:xlink="http://www.w3.org/1999/xlink" lspace="-2pt">{\mathcal{A}}</mml:mpadded>}\limits^{<mml:mpadded xmlns:xlink="http://www.w3.org/1999/xlink" voffset="0">{.}</mml:mpadded>}=-{{\rm{\Gamma }}}_{1}{\mathcal{A}}\,[1+\frac{\zeta |{\mathcal{A}}{|}^{4}}{1+i[(\delta {\omega }_{12}/{{\rm{\Gamma }}}_{2})+|{\mathcal{A}}{|}^{2}]}].$$

Here, $\zeta ={({\gamma }_{{\rm{res}}}/3{\gamma }_{11})}^{2}{{\rm{\Gamma }}}_{2}/{{\rm{\Gamma }}}_{1}$ is the dimensionless characteristic of the strength of the resonant coupling; *ζ* and the ratio *δω*_12_/Γ_2_ fully determine the decay of the mode amplitude ${A}_{1}\,\propto \,|{\mathcal{A}}|$ in dimensionless time Γ_1_ *t*.

It is seen from Eq. (4) that the backaction from the fast-decaying mode 2 leads to a nonexponential decay of the amplitude *A*_1_. The coupling induced decay rate can be understood in terms of the standard Fermi golden rule^40^. It is quadratic in the coupling parameter *γ*_res_ and is proportional to the “density of states” ${{\rm{\Gamma }}}_{2}/[{{\rm{\Gamma }}}_{2}^{2}+{({\omega }_{2}-3{\omega }_{1}^{{\rm{eff}}})}^{2}]$ of the effective reservoir provided by mode 2 at triple the frequency of mode 1, ${\omega }_{1}^{{\rm{eff}}}={\omega }_{1}+{{\rm{\Gamma }}}_{2}|{\mathcal{A}}{|}^{2}$.

**Figure 2:**
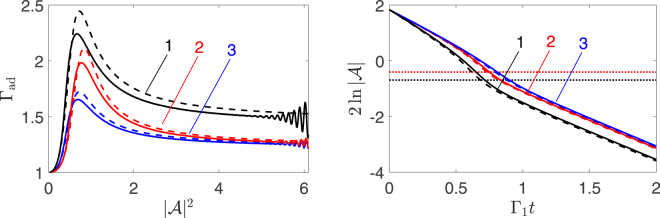
Resonant peak of the instantaneous decay rate. Left panel: The dependence of the normalized effective instantaneous decay rate ${{\rm{\Gamma }}}_{{\rm{ad}}}=-{{\rm{\Gamma }}}_{1}^{-1}(d/dt)\,\mathrm{ln}\,|{\mathcal{A}}|$ on the scaled vibration amplitude $|{\mathcal{A}}|$ of mode 1 in the adiabatic regime of fast decaying mode 2. Right panel: The amplitude of mode 1 in scaled time Γ_1_ *t*; the horizontal dotted lines indicate the amplitude where the resonance occurs for the respective parameter values, $3{\omega }_{1}^{{\rm{eff}}}={\omega }_{2}$ (the black and blue dotted lines overlap). Solid lines are from numerical solution of Eqs (2), and dashed lines are the adiabatic approximation (4). Black, red, and blue curves in both panels (curves 1, 2, and 3) refer to *ζ* = 4, *δω*_12_/Γ_2_ = −1.5; *ζ* = 2, *δω*_12_/Γ_2_ = −2; and *ζ* = 2, *δω*_12_/Γ_2_ = −1.5, respectively; Γ_2_/Γ_1_ = 50. The oscillations at large $|{\mathcal{A}}{|}^{2}$ in the left panel are the effect of the initial conditions where the modes approach the adiabatic regime.

Equation (4) gives in the explicit form the instantaneous decay rate $(d/dt)\,\mathrm{ln}\,|{\mathcal{A}}|$ as function of the mode-1 amplitude. It is illustrated in Fig. 2. The rate displays a resonant peak for 3*ω*_1eff_ = *ω*_2_, in agreement with the Fermi golden rule. The height of the peak increases with the increasing coupling strength *ζ* and with the increasing frequency detuning $|\delta {\omega }_{12}|$. Interestingly, the rate becomes amplitude-independent not only for small amplitudes, where it approaches the linear-decay value Γ_1_, but also for $|{\mathcal{A}}{|}^{2}\,\gg \,|\delta {\omega }_{12}|/{{\rm{\Gamma }}}_{2}$, where it becomes $\approx {{\rm{\Gamma }}}_{1}\mathrm{(1}+\zeta \mathrm{/9)}$. The unusual quasi-linear large-$|{\mathcal{A}}{|}^{2}$ behavior is a consequence of the strong increase of the mode coupling with the increasing vibration amplitude. It is clear from (4) and also seen from Fig. 2 that a peak of the decay rate as function of amplitude corresponds to a kink on the time dependence of the amplitude, where the decay rate quickly changes between its values for large and small amplitudes.

### Nonlinear resonance for weak dissipation

The nontrivial aspects of the dynamics of resonating modes also come forth in the opposite limit, in which the mode relaxation rates are small compared to the rate of the inter-mode energy exchange at resonance. In nano-mechanical systems, all these rates are typically much smaller than the vibration frequencies *ω*_1,2_. One can then think of the evolution as decay of the vibration amplitudes, accompanied by their oscillations that result from the intermode energy exchange^41^.

The mode dynamics in the absence of decay is interesting on its own and needs to be understood first. The conservative system is characterized by two conserved quantities. The first is the effective energy in the rotating frame and is given by function *H*_RWA_ (3). The second is an analog of the Manley-Rowe invariant in nonlinear optics^42^ and has the form $M=I+{Q}_{2}^{2}+{P}_{2}^{2}$ for the considered 3:1 resonance, where $I=({Q}_{1}^{2}+{P}_{1}^{2}\mathrm{)/3}\equiv {\omega }_{1}{A}_{1}^{2}\mathrm{/3}$ is the scaled squared amplitude of mode 1. The dynamics is conveniently described by two canonically conjugate variables *I* and *ϕ*, where ${\varphi }\,=\,3\,{\rm{\arg }}({Q}_{1}-i{P}_{1})-{\rm{\arg }}({Q}_{2}-i{P}_{2})$ is the mode phase difference. In dimensionless time $\tau =3{\gamma }_{{\rm{res}}}t\mathrm{/4}{\omega }_{1}^{2}$ the Hamiltonian equations for these variables read

5 $${\partial }_{\tau }I=-{\partial }_{{\varphi }}h,\quad \,{\partial }_{\tau }{\varphi }={\partial }_{I}h;\quad \quad h(I,{\varphi })=I\delta {{\rm{\Omega }}}_{12}+\frac{1}{2}{\mu }_{1}{I}^{2}+\frac{1}{2}{\mu }_{2}{(M-I)}^{2}+{I}^{\mathrm{3/2}}{(M-I)}^{\mathrm{1/2}}\,\cos \,{\varphi }\mathrm{.}$$

Here $\delta {{\rm{\Omega }}}_{12}=4{\omega }_{1}^{2}\delta {\omega }_{12}\mathrm{/3}{\gamma }_{{\rm{res}}},\,{\mu }_{1}=9{\gamma }_{1}\mathrm{/2}{\gamma }_{{\rm{res}}}$, and ${\mu }_{2}={\gamma }_{2}\mathrm{/18}{\gamma }_{{\rm{res}}}$ are the dimensionles parameters that determine the dynamics; for brevity, we have set *γ*_res_ > 0.

The effective Hamiltonian $h\propto {H}_{{\rm{RWA}}}$ is singular for $I\to M$ and $I\to 0$, i.e., in the range where the amplitude of one of the modes goes to zero. This feature is generic for nonlinear resonance in weakly nonlinear oscillators. It leads to the unusual behavior discussed below.

**Figure 3:**
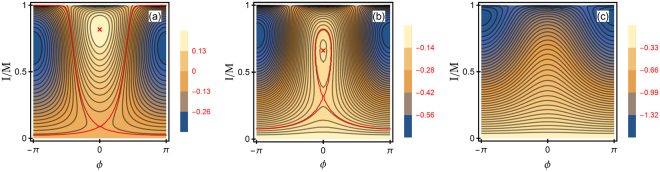
Phase trajectories of the resonating modes in the absence of decay. Motion along the trajectories (thin black lines) corresponds to oscillations in time of the scaled squared amplitudes *I* and *M* − *I* of modes 1 and 2, respectively, and of the mode phase difference *ϕ*. The pattern is periodic in *ϕ* with period 2*π*. The values of the Hamiltonian *h*, Eq. (5), are color-coded, *h* is constant on a trajectory. The scaled parameters of the Duffing nonlinearity are *μ*_1_ = 1, *μ*_2_ = 0, the scaled frequency detuning in (**a**)–(**c**) is $\delta {{\rm{\Omega }}}_{12}/M=-\mathrm{0.5,}-\mathrm{0.9,}-2$. The red lines show the separatrices that go into/out of the saddle point. The red crosses in (**a**) and (**b**) mark the extrema of *h* at *ϕ* = 0. The pattern of the trajectories is the same for $\delta {{\rm{\Omega }}}_{12}\to -\delta {{\rm{\Omega }}}_{12},{\mu }_{1}\to -{\mu }_{1},{\varphi }\to {\varphi }+\pi$.

The dynamics (5) can be mapped onto motion of a particle in a potential well and are described by Jacobi elliptic functions; see Supplemental Material (SM). The qualitative insight into the dynamics comes from the phase portraits shown in Fig. 3(a)–(c). They refer to the case where the Duffing nonlinearity of mode 2 can be disregarded, *μ*_2_ = 0 (a nonzero *μ*_2_ does not change the qualitative picture, see SM). The exact nonlinear resonance $3{\omega }_{1}^{{\rm{eff}}}={\omega }_{2}^{{\rm{eff}}}$ then occurs for $\delta {{\rm{\Omega }}}_{12}=-{\mu }_{1}I$. Therefore we concentrate on the case ${\mu }_{1}\,\delta {{\rm{\Omega }}}_{12} < 0$ where the modes can be tuned in resonance by increasing the amplitude of mode 1.

Shown in Fig. 3 are the lines of constant *h*, which are, essentially, parametric plots of the trajectories *I*(*τ*), *ϕ*(*τ*). The closed loops correspond to oscillations of *I*, *ϕ* about the stationary states where *h*(*I*, *τ*) is maximal or minimal. From Eq. (5), the typical dimensional frequency of these oscillations is $\sim {\gamma }_{{\rm{res}}}/{\omega }_{1}^{2}$. In contrast, on the open trajectories ${\varphi }({\rm{mod}}\,2\pi )$ runs from −*π* to *π*. These trajectories correspond to accumulation of phase *ϕ* in time, which is also accompanied by oscillations of the mode amplitudes. We note that, in the absence of resonant mode coupling, the phase trajectories are just straight horizontal lines, as in this case the amplitudes of the modes are constant on times small compared to the decay time.

The closed trajectories circling around different extrema of *h* are separated from each other and from the open trajectories by separatrices. A peculiar feature of the system is that, in contrast to the “conventional” picture of a phase plane^1^, there are two types of separatrices (Methods). Of particular importance are the separatrices shown by red lines in Fig. 3(a) and (b), which go to/from the saddle point of *h*(*I*, *ϕ*) (SM). This point is a stationary state, ${\partial }_{\tau }I={\partial }_{\tau }{\varphi }=0$. However, rather than circling it, the nearby trajectories approach and then move away, except for the separatrices.

It is important to note the change of the phase portrait from panel (a) to (c) in Fig. 3. With the increasing ratio $|\delta {{\rm{\Omega }}}_{12}|/M$, the saddle point and the extremum of *h* (at *ϕ* = 0 for *μ*_*1*_ > 0) move closer to each other. Ultimately they merge (the saddle-node bifurcation^1^) and disappear. As the extremum disappears, the trajectories that circled it transform into open trajectories.

In the presence of decay, *h* and *M* are no longer conserved. However, they vary in time slowly compared to the oscillation period. One can therefore think of the dynamics as described by Eq. (5) with slowly evolving *h* and *M*. From Eq. (2), time evolution of the Manley-Rowe parameter $M={\omega }_{1}{A}_{1}^{2}\mathrm{/3}+{\omega }_{2}{A}_{2}^{2}$ is described by the equation

6 $$\langle \dot{M}\rangle =-2{{\rm{\Gamma }}}_{2}M+\mathrm{2(}{{\rm{\Gamma }}}_{2}-{{\rm{\Gamma }}}_{1})\langle I\rangle ,$$

where the angular brackets denote time averaging over the period of motion with given *h* and *M*. A similar equation can be written for 〈*h*〉 (Methods).

From Eq. (6) and the condition $M-I\ge 0$, *M* monotonically decreases in time. Physically, this is a consequence of the decrease of the energy of the coupled modes. As *M* decreases, the ratio $|\delta {{\rm{\Omega }}}_{12}|/M$ increases. Therefore Fig. 3(a) to (c) can be thought of as the snapshots of the phase portrait at successive times (disregarding the nonqualitative modification of the phase portrait due to the decay). They refer to the most interesting case where, initially, $|\delta {{\rm{\Omega }}}_{12}|/M$ is small and the system has two well separated centers and a saddle point [Fig. 3(a)]; with increasing time, one of the centers and the saddle point move closer to each other [Fig. 3(b)]; still later, these points merge and disappear [Fig. 3(c)].

**Figure 4:**
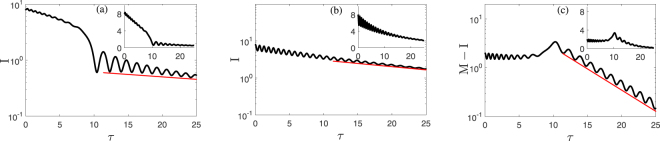
Time evolution of the amplitudes of the modes for small decay rates. The scaled squared amplitudes $I={\omega }_{1}{A}_{1}^{2}\mathrm{/3}$ [panels (a) and (b)] and $M-I={\omega }_{2}{A}_{2}^{2}$ [panel (c)] of modes 1 and 2, respectively, are shown as functions of the scaled time $\tau =3{\gamma }_{{\rm{res}}}t\mathrm{/4}{\omega }_{1}^{2}$. The main figures are on the logarithmic scale, the red lines show the exponential decay of *I* and *M* − *I* in the small-amplitude limit. The insets show the time evolution on the linear scale. The parameters are $\delta {{\rm{\Omega }}}_{12}=-5,\,{\mu }_{1}=1$, and *M*(0) = 10, which corresponds to the parameter values in Fig. 3(a). The initial values of the phase *ϕ*(0) = 0 in panels (a) and (c), *ϕ*(0) = *π* in panel (b), and *I*(0) = 8 correspond to the trajectories that start near the centers at *ϕ* = 0 and *ϕ* = *π* in Fig. 3(a). The decay rates are $4{\omega }_{1}^{2}{{\rm{\Gamma }}}_{1}\mathrm{/3}{\gamma }_{{\rm{res}}}=\mathrm{0.02,}\,{{\rm{\Gamma }}}_{2}/{{\rm{\Gamma }}}_{1}=5$.

From the above arguments, the time evolution of the squared vibration amplitude ${A}_{1}^{2}\propto I$ can follow qualitatively distinct routes depending on the initial conditions. Samples of this evolution are shown in Fig. 4 for two extreme cases, where the initial values of the mode amplitudes and phases are close to different extrema of *h*, i.e., to different centers in Fig. 3 (see SM for an intermediate case). In the presence of dissipation, the closed orbits in Fig. 3 become spirals. As the system moves along a spiral trajectory, *I*(*τ*) oscillates, whereas 〈*I*(*τ*)〉 slowly decays. This behavior is common to the initial portion of the traces in Fig. 4(a),(b).

The functions *I*(*τ*) become qualitatively different as the increasing $|\delta {{\rm{\Omega }}}_{12}|/M$ approaches the value where one of the extrema of *h* disappears (in the considered case, that at *ϕ* = 0 in Fig. 3). Here, the orbit that initially circulated about this extremum dramatically changes, see Fig. 4(a). The value of *I* sharply drops. We find that, after the transient, the system evolves along an open orbit in Fig. 3, that is slightly modified by the dissipation and has a comparatively small *I/M*. The scaled squared amplitude *I* oscillates and 〈*I*〉 decays, but with a different decrement than above the bifurcation point. In contrast, the orbit that started near the other extremum of *h* (at *ϕ* = *π*), keeps oscillating, with 〈*I*〉 decaying smoothly, although the decay is nonexponential, except for small *I*, see Fig. 4(b).

The sharp change of *I* is a consequence of the change of the topology of the phase portrait. It occurs near resonance where $3{\omega }_{1}^{{\rm{eff}}}={\omega }_{2}^{{\rm{eff}}}$ and is not related to the ratio of the mode decay rates (SM). Naturally, it is not described by the method of averaging^43^ (we remind that the averaging here is done in the rotating frame). As seen from Fig. 4(c), the drop of the squared amplitude of mode 1 can be accompanied by the increase of the squared amplitude ${A}_{2}^{2}={\omega }_{2}^{-1}(M-I)$ of mode 2 (Methods).

The nontrivial time evolution of the amplitude of the low-frequency mode in 3:1 resonance was carefully studied in the experiments^14,15^, and through simulations^14^. It was discovered that the mode decay is accompanied by amplitude oscillations. However, their employed methods did not allow revealing the sharp drop of *I*(*τ*) between different regimes of decaying oscillations and the mechanism of this effect, as well as the qualitative difference of the behavior of *I(t)* depending on the initial conditions.

## Discussion

The results of this paper show the rich dynamics of micro- and nanomechanical systems in which vibrational modes experience nonlinear resonance. The features of the dynamics come from the separation of the time scales of the fast vibrations and the comparatively slow evolution of their amplitudes and phases, which are themselves controlled by the interplay of the modes’ nonlinearity, decay, and the nonlinear resonant coupling. These features make the dynamics different from the conventional dynamics of nonlinear resonance and the already complicated dynamics of individual nonlinear modes in the absence of resonance.

Unexpected behavior can be seen by following the mode decay in time, which is a basic tool in the studies of micro- and nano-mechanical systems. Our specific results refer to the decay of the modes that are close to 3:1 resonance, but the revealed behavior is common for internal resonance of weakly damped modes (Methods).

Because of the nonlinear resonance, the decay of vibrational modes becomes strongly non-exponential, with a decay rate that depends on the vibration amplitude, sometimes in a non-monotonic manner. This is a consequence of the amplitude dependencies of the coupling strength and the mode frequencies, which lead to tuning the modes into and out of resonance as their amplitudes vary. The effect is described explicitly in an important case where the high-frequency mode decays much faster than the low-frequency one. Here, the high-frequency mode serves as a thermal reservoir for the low-frequency mode. The results can be understood in terms of an effective transient Purcell effect, or an anomalous nonlinear friction, with a pronounced peak in the dependence of the friction coefficient on the mode amplitude.

A very unusual behavior occurs if the decay rates of the modes are small compared to the appropriately scaled nonlinear coupling. Here, the decay of the vibration amplitude is accompanied by oscillations with a frequency determined by the nonlinear coupling. This frequency is much smaller than the mode frequencies *ω*_1,2_, but can be much higher than the decay rate. Depending on the initial conditions, the oscillations can be qualitatively different. They may smoothly decay along with the mean value of the amplitude. However, unexpectedly, the amplitude can also experience a steep crossover between regimes in which both the magnitude of the oscillations and the mean value of the amplitude are significantly different. Such a jump is a consequence of the change of the topology of the phase portrait in the absence of decay, which is generic for resonating nanomechanical modes.

The features of the nonlinear resonance found in this paper, including the transient Purcell effect and the sharp switching between different regimes of decay, provide insight into existing experimental observations in nano- and micro-mechanical systems and suggest new experiments. The results also suggest new ways of extracting the parameters of the systems and of controlling transient processes in nanomechanics.

## Methods

The calculations in the paper are done for the Duffing nonlinearity parameter *γ*_1_ > 0. In this case the modes are tuned in resonance with increasing amplitude *A*_1_ for *δω*_12_ < 0. A generalization to ${\gamma }_{1} < \mathrm{0,}\,\delta {\omega }_{12} > 0$ is straightforward.

### Resonant nonlinear friction

For Γ_2_ $\gg$ Γ_1_, mode 2 follows mode 1 adiabatically. In the adiabatic approximation, one solves the equation of motion for mode 2 by introducing its complex scaled amplitude ${\mathcal B} =({Q}_{2}-i{P}_{2})\,\exp \,[-3i{\rm{\Phi }}(t)]$ and disregarding in Eq. (2) *d* ${\mathcal B}$ */dt* compared to Γ_2_ ${\mathcal B}$. This results in a linear algebraic equation for ${\mathcal B}$. Its solution gives Eq. (4). Such analysis disregards the frequency shift of mode 2 due to its Duffing nonlinearity, which is justified for large Γ_2_. This explains why Eq. (4) contains *ω*_2_ rather than ${\omega }_{2}^{{\rm{eff}}}$. Equation (4) applies if $|\mathop{<mml:mpadded xmlns:xlink="http://www.w3.org/1999/xlink" lspace="-2pt">{\mathcal{A}}</mml:mpadded>}\limits^{<mml:mpadded xmlns:xlink="http://www.w3.org/1999/xlink" voffset="0">{.}</mml:mpadded>}|\,\ll \,{{\rm{\Gamma }}}_{2}|{\mathcal{A}}|$. This imposes a constraint on the strength of the resonant mode coupling for which the adiabatic approximation holds, $\zeta \,\delta {\omega }_{12}^{2}/{{\rm{\Gamma }}}_{2}^{2}\,\ll \,{{\rm{\Gamma }}}_{2}/{{\rm{\Gamma }}}_{1}$.

The nonexponential decay described by Eq. (4) can be explcitily illustrated for comparatively small amplitudes or strong detuning $|\delta {\omega }_{12}|$, where in (4) ${\omega }_{1}^{{\rm{eff}}}$ can be replaced with *ω*_1_. Then for the scaled squared vibration amplitude $e(t)={[\zeta \mathrm{/(1}+{(\delta {\omega }_{12}/{{\rm{\Gamma }}}_{2})}^{2})]}^{\mathrm{1/2}}|{\mathcal{A}}(t{)|}^{2}$ one obtains

$$e(t)=e\mathrm{(0)}\,\exp \,(-2{{\rm{\Gamma }}}_{1}t\mathrm{)\{1}+{e}^{2}\mathrm{(0)[1}-\exp (-4{{\rm{\Gamma }}}_{1}t{)]\}}^{-\mathrm{1/2}}$$

The decay of *e*(*t*) is faster than exponential, and becomes exponential only for large time, where *e*(*t*) $\ll$ 1. We note the difference of the functional form of *e*(*t*) from the decay for nonresonant nonlinear friction^18,24^.

Along with dissipation, the backaction from the mode coupling leads to a change of the effective vibration frequency, ${\omega }_{1}^{{\rm{eff}}}\to {\omega }_{1}^{{\rm{eff}}}+{\rm{\Delta }}{\omega }_{1}$, which in turn leads to a change of the phase of 𝒜. From Eq. (4), ${\rm{\Delta }}{\omega }_{1}=-{{\rm{\Gamma }}}_{1}\zeta |{\mathcal{A}}{|}^{4}{\rm{Im}}\{{\mathrm{[1}+i\mathrm{(3|}{\mathcal{A}}{|}^{2}+\delta {\omega }_{12}/{{\rm{\Gamma }}}_{2})]}^{-1}\}\mathrm{.}$ The shift Δ*ω*_1_ is a nonlinear counterpart of frequency anti-crossing for strong damping Γ_2_. It is a strongly nonlinear function of the vibration amplitude, which changes sign for $3{\omega }_{1}^{{\rm{eff}}}={\omega }_{2}$. The latter may lead to a kink on the dependence of the overall mode-1 frequency ${\omega }_{1}^{{\rm{eff}}}+{\rm{\Delta }}{\omega }_{1}$ on the scaled amplitude $|{\mathcal{A}}|$.

A resonant peak of the decay rate as function of the mode amplitude emerges also for 2:1 resonance. If *ω*_2_ is close to 2*ω*_1_, the dynamics of resonantly coupled modes in the rotating frame are described by Eqs (2) and (3) with the coupling term $\propto {\gamma }_{{\rm{res}}}$ in *H*_RWA_ replaced with $({\beta }_{{\rm{r}}{\rm{e}}{\rm{s}}}/2{\omega }_{1}){\rm{R}}{\rm{e}}\,[({Q}_{1}-i{P}_{1}{)}^{2}({Q}_{2}+i{P}_{2})]$. For Γ_2_ $\gg$ Γ_1_ the resonant nonlinear friction is described by equation

$$\mathop{<mml:mpadded xmlns:xlink="http://www.w3.org/1999/xlink" lspace="-2pt">{\mathcal{A}}</mml:mpadded>}\limits^{<mml:mpadded xmlns:xlink="http://www.w3.org/1999/xlink" voffset="0">{.}</mml:mpadded>}=-{{\rm{\Gamma }}}_{1}{\mathcal{A}}\,[1+\frac{{{\zeta }^{{\rm{^{\prime} }}}}_{{\rm{r}}{\rm{e}}{\rm{s}}}|{\mathcal{A}}{|}^{2}}{1+i(2{\omega }_{1}^{{\rm{e}}{\rm{f}}{\rm{f}}}-{\omega }_{2})/{{\rm{\Gamma }}}_{2}}]$$

with ${\zeta ^{\prime} }_{{\rm{res}}}=4{\beta }_{{\rm{res}}}^{2}\mathrm{/3}{\gamma }_{1}$. As seen from this equation, the decay for 2:1 resonance is similar to that for 3:1 resonance. We note, however, that in this case the decay rate approaches its linear value at both small and large amplitudes.

### The decay-free dynamics

The conservative dynamics of the coupled modes in the RWA differs from the conventional picture of nonlinear resonance in the action-angle variables^23^. In Eq. (5), the effective “action” variable *I* is limited, $0\le I\le M$. For $I\to M$ the dynamics becomes singular, $|{\partial }_{\tau }{\varphi }|\,\to \,\infty$. The points (${\varphi }=\mathrm{(2}n+\mathrm{1)}\pi \mathrm{/2,}\,I\to M$) separate regions where ${\partial }_{\tau }{\varphi }$ has opposite signs, and the nearby trajectories move in opposite directions along the *ϕ*-axis. In particular, they can spin around different centers, as in Fig. 3. There is no slowing down near these points. On a trajectory that at *τ* = 0 goes through a point ${{\varphi }}_{0}=\pi \mathrm{/2}-{\varepsilon }_{{\varphi }},\,{I}_{0}=M\mathrm{(1}-{\varepsilon }_{I})$ with small $|{\varepsilon }_{{\varphi }}|$ and *ε*_*I*_, we have $\tan \,{\varphi }(\tau )\approx {\varepsilon }_{{\varphi }}^{-1}-M\tau \mathrm{/(2}{\varepsilon }_{I}^{\mathrm{1/2}}{\varepsilon }_{{\varphi }})$ for $\tau \lesssim {\varepsilon }_{I}^{\mathrm{1/2}}/M$. The time ${\varepsilon }_{I}^{\mathrm{1/2}}/M$ is the typical dimensionless time to go over the phase interval *π* for small *M* − *I*.

In the presence of weak dissipation, the effective Hamiltonian *h* is no longer conserved and ultimately decays to zero, along with the Manley-Rowe invariant *M*. From Eq. (2), for *μ*_2_ = 0 the period-averaged rate at which *h* changes is

7 $$\langle \dot{h}\rangle =-2{{\rm{\Gamma }}}_{1}\langle I\dot{{\varphi }}\rangle +\frac{1}{2}\langle \frac{{I}^{\mathrm{3/2}}\dot{M}}{{(M-I)}^{\mathrm{1/2}}}\,\cos \,{\varphi }\rangle $$

(note that the derivatives here and in Eq. (6) are taken with respect to dimensional time *t*). Equations (6) and (7) apply for *h* sufficiently far from from its saddle-point value, so that the period of motion with constant *h* and *M* is small compared to the reciprocal decay rates ${{\rm{\Gamma }}}_{\mathrm{1,2}}^{-1}$. In contrast to the Manley-Rowe invariant *M*, the evolution of *h* may be nonmonotonic.

The anomalous behavior of the squared amplitude of mode 2, ${A}_{2}^{2}={\omega }_{2}^{-1}(M-I)$, in the presence of dissipation shown in Fig. 4(c), can be understood from Eq. (6). If Γ_2_ $\gg$ Γ_1_ and 〈*I*〉 varies on a time scale longer than 1/Γ_2_, as in the initial section of Fig. 4(a), the quasistationary solution of Eq. (6) is $M-\langle I\rangle \approx -\mathrm{(2}{{\rm{\Gamma }}}_{1}\langle I\rangle +\langle \dot{I}\rangle \mathrm{)/2}{{\rm{\Gamma }}}_{2}$. Therefore $\langle {A}_{2}^{2}\rangle$ remains small and weakly varies before the drop of *I*. The drop of *I* is associated with a fast switching between different quasiperiodic orbits. Therefore *M* − *I* can increase where *I* drops. Physically, this increase corresponds to a resonant energy transfer from mode 1 to mode 2. This process is not described by the averaging method and by the quasistationary solution, there is no time scale separation between oscillations with given *h* and the decay.

## Electronic supplementary material

Supplementary Material
