# Supplementary material for: Anomalous Decay of Nanomechanical Modes Going Through Nonlinear Resonance
Source: Sci Rep. 2017 Dec 22;7:18091. doi: 10.1038/s41598-017-17184-6 (PMC5741793; doi:10.1038/s41598-017-17184-6)
Supplement: Supplementary file 1 — Supplementary Material [file 41598_2017_17184_MOESM1_ESM.pdf]

SUPPLEMENTAL MATERIAL:

Anomalous Decay of Nanomechanical Modes Going Through Nonlinear Resonance

O. Shoshani, S. W. Shaw, and M. I. Dykman

The effective Hamiltonian in the absence of dissipation. Fig. 1 shows the effective Hamiltonian of the resonating nonlinearly coupled modes $h(I,\phi)$ as function of the scaled squared amplitude of mode 1, $I = \omega_1 A_1^2/3$, and the phase difference between the modes ϕ. The Hamiltonian is given by Eq. (5) of the main text, which we reproduce here for completeness,

$$h(I,\phi) = I\delta\Omega_{12} + \frac{1}{2}\mu_1 I^2 + \frac{1}{2}\mu_2 (M-I)^2 + I^{3/2}(M-I)^{1/2}\cos\phi \tag{1}$$

It is clear from Eq. (1) that the internal nonlinearity of mode 2, which is characterized by the parameter μ_2 can be incorporated into the analysis by renormalizing

$$\mu_1 \to \mu_1 + \mu_2,\ \delta\Omega_{12} \to \delta\Omega_{12} - \mu_2 M,\ h \to h + \frac{1}{2}\mu_2 M^2 \tag{2}$$

Equation (2) shows that much of the qualitative features of the dynamics can be captured by setting $\mu_2 = 0$, as it is done in the main text and in the analysis below.

For the chosen signs of the parameters in Fig. 1, $\delta\Omega_{12} < 0$ and $\mu_1 > 0$, function $h(I,\phi)$ has a minimum for $\phi(\mathrm{mod}\,2\pi) = \pi$ and a maximum for $\phi(\mathrm{mod}\,2\pi) = 0$. Also clearly seen is a saddle point h_S for $\phi_S(\mathrm{mod}\,2\pi) = 0$ and $(I/M)_S \approx 0.18$: the curvature at the saddle point is positive along the I-axis and negative along the ϕ-axis.

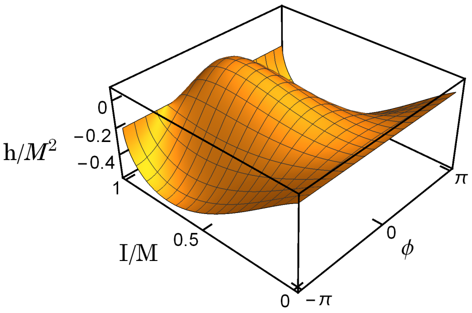

Figure 1. The scaled Hamiltonian $h(I,\phi)/M^2$ of the coupled modes in the absence of decay. The scaling factor M is related to the mode amplitudes $A_{1,2}$ as $M = \omega_2 A_2^2 + I$, with $I = \omega_1 A_1^2/3$. The plot refers to the following parameter values in Eq. (1): $\delta\Omega_{12}/M = -0.7, \mu_1 = 1, \mu_2 = 0$.

The trajectories in Fig. 3 of the main text are the cross-sections of the surface $h(I,\phi)$ by the planes $h =$ const. Figure 1 shows that the cross-sections with the values of h near the minimum of $h(I,\phi)$ are closed contours (the closed loops in the blue area in Fig. 3 of the main text). A cross-section for the saddle-point value of the Hamiltonian $h = h_S$ gives a closed loop around the maximum and also an open contour where ϕ varies across the whole range of periodicity $(-\pi,\pi)$, cf. the red trajectory in Figs. 3(a),(b) of the main text. The cross-section for the values of h between h_S and the maximum of $h(I,\phi)$ represent closed contours centered around the maximum, and open contours, as seen in Fig. 3 of the main text.

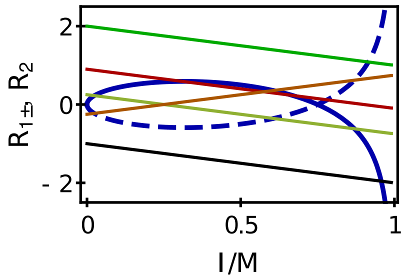

Figure 2. Change of the topology of the phase portrait with the varying Manley-Rowe parameter. The functions $R_{1\pm}(I/M)$, Eq. (3), are shown by dark blue lines (the signs "+" and "-" refer to the solid and dashed line, respectively). The straight lines with negative slope give $R_2(I/M)$ for $\mu_1 = 1$. The parameter $\delta\Omega_{12}/M$ from top to bottom is $-2, -0.9, -0.25, 1$ (the green, red, light green, and black lines, respectively). The straight brown line with positive slope gives R_2 for $\mu_1 = -1$ and $\delta\Omega_{12}/M = -0.25$. The intersections of the straight lines with the solid and dashed blue lines give the values of I/M at the stationary states for $\phi = 0$ and $\phi = \pi$, respectively. The number of intersections, and thus the topology of the phase portrait, change with varying $\delta\Omega_{12}/M$

The change of the topology of the phase portrait due to decay. An important insight into the dynamics in the presence of decay comes from Fig. 2, which complements the previous analysis. This figure allows finding the location of the stationary states of the Hamiltonian dynamics $\partial_I h = \partial_\phi h = 0$ and following their evolution in time due to decay. Such evolution includes the coalescence of the saddle point and an extremum of $h(I,\phi)$ (the saddle-node bifurcation), which leads to the dramatic change of the mode amplitudes discussed in the main text.

From Eq. (1), the stationary states (the extrema and the saddle point) of $h(I,\phi)$ are located at $\phi(\mathrm{mod}\, 2\pi) = 0$ and $\phi(\mathrm{mod}\, 2\pi) = \pi$. Their positions along the I-axis are given by equation $\partial_I h = 0$ evaluated for $\phi = 0$ and $\phi = \pi$. This equation can be conveniently written as $R_{1\pm}(I/M) = R_2(I/M)$, where

$$R_{1\pm}(x) = \pm\frac{(3-4x)\sqrt{x}}{2\sqrt{1-x}}, \quad R_2(x) = -\frac{\delta\Omega_{12}}{M} - \mu_1 x \tag{3}$$

Functions $R_{1\pm}(I/M), R_2(I/M)$ are shown in Fig. 2. Function $R_{1\pm}$ has no parameters. In contrast, the slope of $R_2(I/M)$ depends on μ_1, whereas $R_2(0)$ is given by $-\delta\Omega_{12}/M$. It is seen from the figure that the number of intersections of $R_{1\pm}$ and R_2, i.e., the number of the stationary states of the Hamiltonian system, sensitively depends on $\delta\Omega_{12}/M$ and μ_1. The system has three stationary states for not too large $|\delta\Omega_{12}|/M$, but as this parameter increases beyond a critical value (which depends on μ_1), there remains only one stationary state. The critical value of $|\delta\Omega_{12}|/M$ determines the point where the corresponding extremum of $h(I,\phi)$ merges with the saddle and disappears. Respectively, the topology of the phase portrait of the Hamiltonian system described by Eq. (1) changes from that in Figs. 3(a) and (b) of the main text to that in Fig. 3(c).

As explained in the main text, the change of the number of the stationary states at the saddle-node bifurcation can lead to a dramatic change of the time dependence of the scaled vibration amplitude I. The fact that, due to decay, the Manley-Rowe invariant M monotonically decreases shows that the ratio $|\delta\Omega_{12}|/M$ monotonically increases due to decay. This corresponds to moving the straight lines in Fig. 2 up (for $\delta\Omega_{12} < 0$) or down (for $\delta\Omega_{12} > 0$). Therefore decay invariably leads to the saddle-node bifurcation. This shows that the sharp drop of the scaled amplitude of mode 1 I is a generic effect, provided the system is initially close to the center that disappears at the bifurcation.

In Fig. 3(a) and (b) we show the time evolution of the scaled squared amplitudes of modes 1 and 2, I and $M-I$, for the same parameter values as in Fig. 4 of the main text, but for the initial point close to the separatrix. The behavior of I and $M-I$ in this case is similar to that in Fig. 4 (a) and (c) of the main text, respectively, except that the jump in I that occurs near the saddle-node bifurcation is less pronounced. For the initial phase value $\phi(0) = \pi/2$

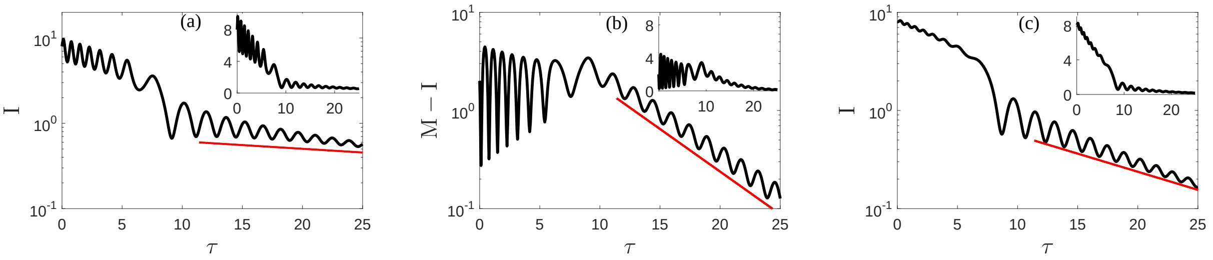

Figure 3. **Time evolution of the amplitudes of the modes for small decay rates**. The scaled squared amplitudes $I = \omega_1 A_1^2/3$ [panels (a) and (c)] and $M - I = \omega_2 A_2^2$ [panel (b)] of modes 1 and 2, respectively, are shown as functions of the scaled time $\tau = 3\gamma_{\rm res} t/4\omega_1^2$. The main figures are on the logarithmic scale, the red lines show the exponential decay of I and $M - I$ in the small-amplitude limit. The insets show the time evolution on the linear scale. The parameters are $\delta\Omega_{12} = -5, \mu_1 = 1$, and $M(0) = 10$, which corresponds to the parameter values in Fig. 3(a) of the main text. The initial values of the phase are $\phi(0) = \pi/4$ in panels (a) and (b), and $\phi(0) = 0$ in panel (c); $I(0) = 8$. The value $\phi(0) = \pi/4$ corresponds to a trajectory that starts near the separatrix in Fig. 3(a) of the main text. The decay rates are $4\omega_1^2\Gamma_1/3\gamma_{\rm res} = 0.02, \Gamma_2/\Gamma_1 = 5$ in panels (a) and (b); in panel (c) $4\omega_1^2\Gamma_1/3\gamma_{\rm res} = 0.05$ and $\Gamma_2/\Gamma_1 = 1$.

the behavior becomes similar to that in panel (b) of Fig. 4 of the main text. Figure 3(c) shows that the jump persists if we choose the decay rates to be equal.

Mapping the decay-free dynamics on that of a particle in a potential well.
Time evolution of the scaled squared amplitude I of mode 1 in the absence of decay can be mapped onto the evolution of the coordinate of a particle with a unit mass, which oscillates in a potential well. These oscillations are described by equation

$$\frac{d^2 I}{d\tau^2} = -\frac{\partial U_{\rm eff}}{\partial I}, \qquad U_{\rm eff}(I) = -\frac{1}{2} I^3 (M - I) + \frac{1}{2}\left[h - \delta\Omega_{12} I - \frac{1}{2}\mu_1 I^2 - \frac{1}{2}\mu_2 (M - I)^2\right]^2, \tag{4}$$

where h is the value of the Hamiltonian (1). Equation (4) is derived from Eq. (1) and the Hamiltonian equations of motion for I and ϕ, Eq. (5) of the main text.

The potential $U_{\rm eff}$ is a quartic polynomial in I. It can have one well or two wells separated by a local maximum. The centers and the saddle point on the phase portraits in Fig. 3 of the main text correspond to $\partial U_{\rm eff}/\partial I = 0$; note, however, that these points on the phase portrait have different values of h, whereas $U_{\rm eff}(I)$ depends on h as a parameter. It is important that the oscillations of $I(\tau)$ in the potential $U_{\rm eff}(I)$ occur with zero effective total energy, $\frac{1}{2}(dI/d\tau)^2 + U_{\rm eff}(I) = 0$, as again can be seen from Eq. (5) of the main text. Therefore Eq. (4) can be reduced to equation

$$\frac{dI}{d\tau} = \pm[-2U_{\rm eff}(I)]^{1/2}. \tag{5}$$

The sign $\pm$ reflects the change of the sign of $dI/d\tau$ at the turning points.

An immediate consequence of Eqs. (4) and (5) is that the time evolution of the scaled squared amplitude $I(\tau)$ is described by Jacobi elliptic functions[1]. This shows, in particular, that in the case of a double-well potential the frequencies of vibrations in the both wells are equal. One of these vibrations corresponds to motion along a trajectory that circles around a center in Fig. 3(a) and (b) of the main text, whereas the other corresponds to motion along an open trajectory beneath the separatrix loop; both trajectories have the same value of h. The explicit expressions for $I(\tau)$ simplify the calculation of its the period-averaged value away from the separatrix.

References

1. Abramowitz, M. & Stegun, I. A. *Handbook of Mathematical Functions with Formulas, Graphs, and Mathematical Table* (Dover Publications, Inc., 1972).
